# Anesthesia and airway managements for emergency removal of esophageal foreign body in a trisomy 21 patient with mental retardation and predicted difficult airway: A case report

**DOI:** 10.1097/MD.0000000000023710

**Published:** 2020-12-18

**Authors:** Wei Wei, Huan-Rong Qiu, Hai-Xia Wang, Fu-Shan Xue

**Affiliations:** Department of Anesthesiology, Beijing Friendship Hospital, Capital Medical University, Beijing, People's Republic of China.

**Keywords:** airway safety, anesthesia, esophageal foreign body, mental retardation, trisomy 21 syndrome

## Abstract

**Introduction:**

The typical manifestations of patients with a trisomy 21 syndrome are mental retardation and anatomical deformities of face and neck. In the available literature, all case reports regarding anesthetic management of mentally retarded patients have focused on elective surgeries. There is no report regarding anesthetic management of mentally retarded patients undergoing emergency surgery.

**Patient concerns:**

A 47-year-old woman with a mental retardation grade 2 by trisomy 21 syndrome suffered from an esophageal foreign body for 3 days and needed emergency removal of esophageal foreign body. The patient had a poor cooperation and obvious anatomical abnormalities of head and neck.

**Diagnoses:**

Difficult anesthesia and airway managements for emergency removal of esophageal foreign bodies in a trisomy 21patients with mental retardation and predicted difficult airways.

**Interventions:**

Combined use of an intubating supraglottic airway and the flexible bronchoscope-guided intubation after intravenous anesthesia induction.

**Outcomes:**

Effective airway was safely established and an esophageal foreign body was successfully removed by rigid esophagoscopy under anesthesia. The patient recovered smoothly without any complication.

**Lessons subsections as per style:**

When general anesthesia and emergency airway management are required in the patients with mental retardation and predicted difficult airways, a combination of the supraglottic airway and the flexible bronchoscope maybe a safe and useful choice for airway control.

## 1 Introduction

Removal of an esophageal foreign body in adult patients is generally performed under procedure sedation with propofol combined with a small amount of opioid drugs and dexmedetomidine.^[1]^ As the typical manifestations of patients with a trisomy 21 syndrome are mental retardation and anatomical deformities of face and neck, they often have a poor cooperation and a predicted difficult airway. For such patients, procedure sedation is evidently inappropriate anesthetic choice.^[2]^ In the available literature, all case reports regarding anesthetic management of mentally retarded patients have focused on elective surgeries,^[3,4]^ for which adequate preparation and assessment can be performed before anesthesia. For mentally retarded patients requiring emergency surgery, however, safe anesthesia and airway managements are major challenges for anesthesiologists, because adequate preoperative assessment and preparation may be difficult. Especially, there is no consensus about how to quickly establish safe airway in such patients who have different risk factors of difficult airways. In this article, we firstly reported our experience about anesthesia and airway managements of a trisomy 21 patient with mental retardation and predicted difficult airways undergoing emergency removal of an esophageal foreign body.

## 2 Case presentation

This publication of this case was approved by the Ethics Committee of Beijing Friendship Hospital and written consent was obtained from her parents for the purpose of publication of case details and images.

A 47-year-old woman with a known trisomy 21 syndrome was about 155 cm tall and weighed about 80 kg. She was diagnosed with a mental retardation grade 2 and had an intelligence quotient of 23. In this time, she was admitted to hospital due to an esophageal foreign body for 3 days. The foreign body, a Chinese date seed, was stuck at the sternal angle level and was suspected to puncture the esophagus and compress trachea, causing moderate wheezing symptoms. For this reason, an urgent procedure to remove the foreign body was required.

### 2.1 Preoperative rapid assessment

**Figure 1:**
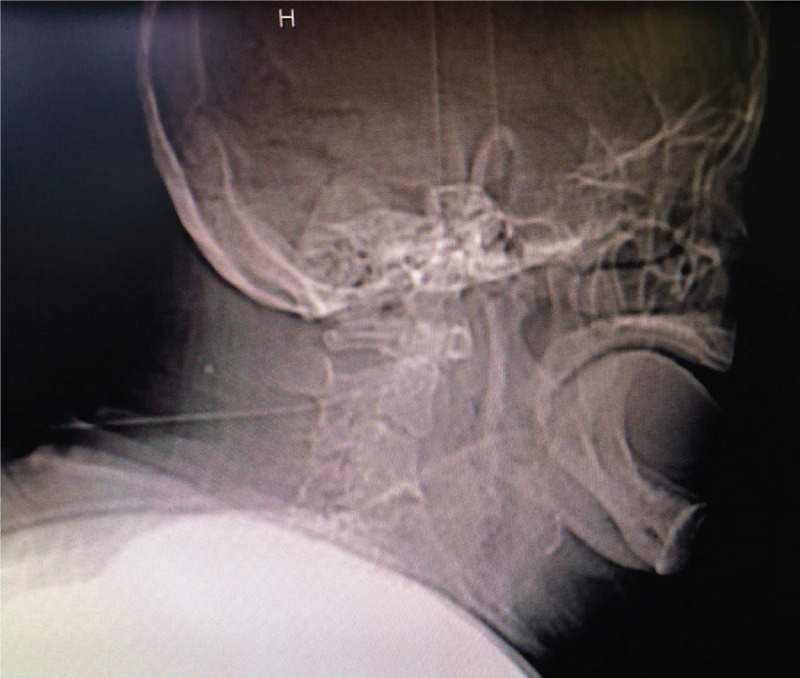
The CT image of patient's head and neck.

The patient was obese with a body mass index of 33.2, was well unable to obey orders and did not cooperate with the examination due to mental retardation. Her tongue was hypertrophic and prominent in the oral cavity. Due to the deformed jaw, mouth opening was significantly limited, with an approximately 3 cm of inter-dental interval. The neck activity was also limited and the width of the chin was only about 4 to 5 cm. The computed tomography images showed thick neck, small jaw and bad cervical alignment (Fig. 1). In the view of these risk factors, difficult airways were predicted.

### 2.2 Airway proposal

Because patient did not cooperate, general anesthesia was needed before the airway was established. Given that patient had a high risk of difficult airway managements after anesthesia induction, and a possibility for the occurrence of “can’t intubate, can’t oxygenate (CICO)” situation after failed intubation, according to the current difficult airway management guideline of the American Society of Anesthesiologists,^[5]^ it was planned to firstly insert an intubating laryngeal mask airway (LMA) with general anesthesia to quickly establish an effective airway and then perform tracheal intubation through the intubating LMA using the flexible fiberscope. After tracheal intubation, the LMA was removed to provide the access to the esophagoscopy and intraoperative ventilation was maintained via tracheal tube.

### 2.3 Anesthesia and airway management

**Figure 2:**
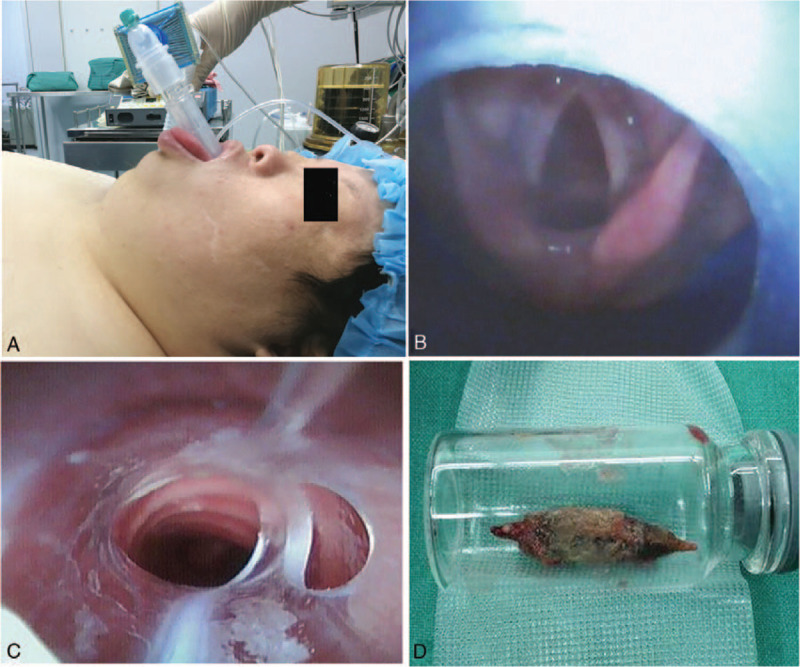
The airway management process and removed foreign body. A: An intubating laryngeal mask airway (LMA) in placement; B: The complete glottic opening was viewed by a flexible video bronchoscope inserted into the upper airway via the intubating LMA; C: A tracheal tube was inserted into the middle trachea under guidance of a flexible video bronchoscope; D: A Chinese date seed removed from the esophagus.

After entering the operating room, patient was sitting and routine monitor including noninvasive blood pressure, electrocardiogram and pulse oxygen saturation was applied. After a venous access was established, anesthesia was induced with intravenous sulfentanil 0.1 μg/kg, propofol 1.5 mg/kg and rocuronium 0.5 mg/kg. After the loss of consciousness, she was placed in a supine position and a thin shoulder pad was placed under the shoulders. The facemask ventilation was attempted with the jaw thrust. After effective facemask ventilation was confirmed and adequate preoxygenation was obtained, a 3-size intubating LMA (Blockbuster laryngeal mask, Tuoren Medical, Henan, China) was inserted (Fig. 2A) and the quality of LMA ventilation was evaluated by observing chest wall movement, auscultation, and capnography. Once adequate ventilation was obtained via the LMA and the correct placement of intubating LMA was determined with the flexible video bronchoscope (OD 3.8 mm, UE Medical Company, Zhejiang, China) (Fig. 2B), the flexible video bronchoscope-guided intubation via the intubating LMA was attempted. A 7-size tracheal tube was successfully inserted into the trachea on the first attempt (Fig. 2C). After successful intubation, the intubating LMA was removed through the tracheal tube, anesthesia was maintained with continuous intravenous infusion of propofol at a rate of 6 mg/kg/h and mechanical ventilation was carried out during the procedure.

### 2.4 Surgical procedure

A Chinese date seed was successfully removed from the esophagus under rigid esophagoscopy (Fig. 2D) and procedure lasted approximately 40 minute. The patient's vital signs were stable during the procedure. After the completion of procedure, anesthesia was discontinued. When spontaneous breathing and consciousness were fully restored, the trachea was extubated and the patient was returned to the ward after a 20-min observation in the PACU. The patient recovered smoothly without any complication and was discharged at the second postoperative day.

## 3 Discussion

As mentally retarded patients are often prone to eating disorders, incarceration may occur when an under-chewed piece of food, nut, or animal bone enters the esophagus.^[2]^ Thus, mental retardation accounts for a large proportion of patients with esophageal foreign bodies. Because patients with mental retardation are unable to provide clear main complaints, esophageal foreign bodies can often not be removed timely. Even the complications by long implantation of esophageal foreign bodies have occurred on admission. Furthermore, incarcerated foreign bodies that often occur in mentally retarded patients may cause serious complications, such as esophagotracheal fistula, aortic rupture, and mediastinal infection.^[6]^ For these reasons, the removal of an esophageal foreign body is often an emergency procedure in mentally retarded patients. In contrast, most of esophageal foreign bodies in normal adult patients may be electively removed because of timely admission and diagnosis.

Emergency removal of esophageal foreign bodies is generally performed with a rigid esophagoscope, which is believed to perform better than flexible esophagoscope in terms of improving procedure and reducing complications.^[1]^ However, the rigid esophagoscopy requires no body movements of patients during the procedure as body movements can significantly affect the procedure and may cause serious complications.^[7]^ Evidently, patients with mental retardation cannot cooperate with the rigid esophagoscopic procedure under awake or sedation status and general anesthesia is required for the removal of an esophagus foreign body with rigid esophagoscopy.

In the available literature, there has been no clinical study on standardization plans of anesthesia and airway managements for emergency removal of esophageal foreign bodies in mentally retarded patients with a trisomy 21 syndrome, and there are only a few studies or case reports on anesthesia and airway management of patients with other mental handicaps undergoing surgical procedures. For example, Khanna et al^[4]^ reviewed their experience of using general anesthesia in the patients with Sturge-Weber syndrome and recommended that for mentally retarded patients undergoing general anesthesia, the supraglottic airway (SGA) should be used for airway control, reducing the hemodynamic responses by tracheal intubation. Similarly, in mentally retarded children with Sturge-Weber syndrome who underwent vitreous surgery under general anesthesia, Khanna and Roodneshin^[8]^ also applied the LMA to establish the artificial airway during surgery. Furthermore, they believed that the LMA not only reduced airway stimulation, but also avoided tracheal intubation. Packiasabapathy et al^[3]^ and Suzuki et al^[9]^ find that in patients with congenital mental disorders, difficult airways are often caused by anatomical abnormalities, which makes anesthesiologists arduous to find glottis. Most important, all of above reports suggest that the LMA can establish the safe airway without a need of glottic exposure, and is a safe airway solution for mentally retarded patients undergoing general anesthesia, especially for mentally retarded patients with known or predicted difficult airways.^[3,4,8,9]^

The specific methods of general anesthesia for the removal of esophageal foreign body reported in the available literatures are significantly different,^[10–12]^ but the main principles are basically the same, namely; providing sufficient depth of anesthesia to inhibit upper airway reflexes, maintain hemodynamic stability and restrain patient's body movements. However, all general anesthetics can lead to significant respiratory inhibition. After patient's consciousness disappears, moreover, posterior dropping of the tongue base and collapse of the oropharyngeal cavity may cause upper airway obstruction and inadequate ventilation, especially for patient with anatomical deformities of the upper airway. When the removal of esophagus foreign bodies is performed under general anesthesia, thus, ensuring the airway safety and adequate ventilation with artificial airways is a priority, especially when emergency procedure is performed.^[11,13]^

Other than mental retardation, patients with a trisomy 21 syndrome are often complicated with the known anatomical abnormalities of head and neck that can cause difficult airways, such as micrognathia, neck stiffness and tongue hypertrophy shown in our patient. The awake intubation is the safest scheme to manage the known or predicted difficult airway,^[5]^ but it is impracticable for patients with mental retardation. Furthermore, sevoflurane inhalational induction with spontaneous breathing is 1 of the recommended methods for anesthetic management in adult and pediatric difficult airways.^[14]^ However, this method requires a long induction time to achieve adequate depth of anesthesia for airway instrumentation^[15]^ and can result in the risks of airway spasm and aspiration during initial induction in patients undergoing emergency procedure.^[16]^ Additionally, it is reported that patients with a trisomy 21 syndrome may develop significant bradycardia during inhalational induction with sevoflurane.^[17]^ In this patient, thus, rapid intravenous anesthesia induction was selected.

When intravenous anesthesia induction was performed in the patients with a known or predicted difficult airway, an important thing is to ensure that airway can be safely controlled after anesthesia induction, especially for when difficult facemask ventilation occurs and intubation attempt fails. The available evidence indicates that the SGA, such as the LMA, is an effective device for management of difficult facemask ventilation and failed intubation.^[18]^ Specially, the intubating SGA can not only maintain the effective ventilation, but also can provide a safe approach for subsequent intubation. Furthermore, an additional gastric drainage channel of second generation SGA, such as Blockbuster laryngeal mask used in our patient, may decrease the risk of aspiration during anesthesia induction.^[19,20]^ However, as the removal of esophageal foreign bodies requires an upper airway shared between the surgeon and the anesthesiologist during the procedure, an placed SGA can obstruct the surgeon’ access to the esophagus and make the esophagoscopy impossible.^[11,13]^ After effective airway is established with the SGA, thus, it is necessary to replace the SGA with tracheal intubation. A main advantage of flexible video bronchoscope-guided intubation using the SGA as a conduit is the ability to allow unhurried video airway instrumentation while maintaining continuous ventilation through a dedicated airway. Even if the flexible video bronchoscope-guided intubation fails, the placed SGA can also maintain effective ventilation, avoiding the occurrence of a “CICO” situation.^[21]^

Certainly, the removal of an esophageal foreign body in a mentally retarded patient may be electively carried out, if his or her family member or guardian can provide a clear history and clinicians ensure the occurrence of no complication associated with the esophageal foreign body. In this situation, adequate preoperative evaluation and preparation are often allowed before the procedure. In performing preoperative airway evaluation, anatomic variation and limitation of joint movement, which may be associated with difficult intubation or difficult mask ventilation, should be identified by overall examinations.^[22]^ Any history of previous airway management should also be reviewed, including prior anesthetic records. If preoperative evaluation and preparation ensure that a safe airway can be immediately established in the situations of airway obstruction and severe respiratory distress, elective removal of an esophageal foreign body may be performed under general anesthesia with inhalation or intravenous agents without tracheal intubation.^[11,12]^ As a general rule, however, spontaneous ventilation should not be abolished during anesthesia induction and maintainence.^[5]^ During the procedure, moreover, appropriate attention should be directed to the attenuation of the tachycardic and hypertensive responses to esophagoscopy. If preoperative evaluation predicts that airway management or removal of an esophageal foreign body is difficult,^[6,11]^ anesthesiologist should proceed with caution and above-mentioned plans for anesthesia and airway management should be recommended.

In summary, when emergency removal of esophagus foreign bodies is performed under general anesthesia in a trisomy 21 patient with mental retardation and predicted difficult airways, combined use of SGA and flexible bronchoscope-guided intubation maybe a safe and useful method for airway control.

## Author contributions

WW, HRQ, HXW and FSX performed anesthesia and airway management; WW and FSX drafted the manuscript and made the final revision. All authors contributed to data analysis, drafting or revising the article, gave final approval of the version to be published, and agree to be accountable for all aspects of the work.

**Conceptualization:** Wei Wei, Huan-Rong Qiu, Hai-Xia Wang, Fu-Shan Xue.

**Data curation:** Wei Wei, Huan-Rong Qiu, Hai-Xia Wang.

**Investigation:** Wei Wei.

**Methodology:** Wei Wei, Huan-Rong Qiu, Fu-Shan Xue.

**Supervision:** Fu-Shan Xue.

**Validation:** Huan-Rong Qiu, Hai-Xia Wang.

**Writing – original draft:** Wei Wei, Hai-Xia Wang.

**Writing – review & editing:** Fu-Shan Xue.
